# Prosaposin hyperglycosylation: a novel tumor immune escape mechanism and implications for cancer immunotherapy

**DOI:** 10.1038/s41392-024-01877-2

**Published:** 2024-07-10

**Authors:** Matthias Peipp, Diana Dudziak, Christian Kellner

**Affiliations:** 1 Division of Antibody-Based Immunotherapy, Department of Medicine II, Christian-Albrechts University and University Hospital Schleswig-Holstein, Kiel, Germany; 2 Institute of Immunology, Jena University Hospital, Friedrich-Schiller-University, Jena, Germany; 3 Comprehensive Cancer Center Central Germany Jena/Leipzig, Jena, Germany; 4 Division of Transfusion Medicine, Cell Therapeutics and Haemostaseology, LMU University Hospital, LMU Munich, Munich, Germany

**Keywords:** Antigen processing and presentation, Tumour immunology

In a recent article published in *Science*, Sharma et al. describes the essential role of saposins in cross-presentation of tumor membrane-associated antigens by dendritic cells (DC) to CD8^+^ T cells.^1^ However, antigen cross-presentation, which is fundamental in cancer cell elimination, is hindered in tumor DC by hyperglycosylation of the common saposin precursor prosaposin (pSAP) leading to impaired T cell tumor immunity—yet, the potential of recombinant pSAP to re-establish T cell responses may offer a novel way to counteract immune evasion in a therapeutic approach.

Cytotoxic CD8^+^ T cells play key roles in tumor immune surveillance.^2^ They recognize antigens presented by major histocompatibility complex (MHC) class I and typically require activation by antigen presenting cells (APC).^3^ Efficient T cell priming requires antigen capture and processing, peptide loading onto MHC molecules and provision of co-stimulatory signals. Dying tumor cells are important tumor membrane antigen sources. The cross-presentation of such exogenous antigens on MHC class I is a prerequisite for the initiation of antitumoral CD8^+^ T cell responses.^3^ In this context, type-1 conventional DC (cDC1), which are specialized for cross-presentation of internalized particulate antigens, are crucial. Tumors interfere with T cell priming in multiple ways including loss of immunogenic antigens, inhibition of DC maturation and creation of an immune-suppressive micro-environment.^4^ However, knowledge is still patchy.

**Fig. 1:**
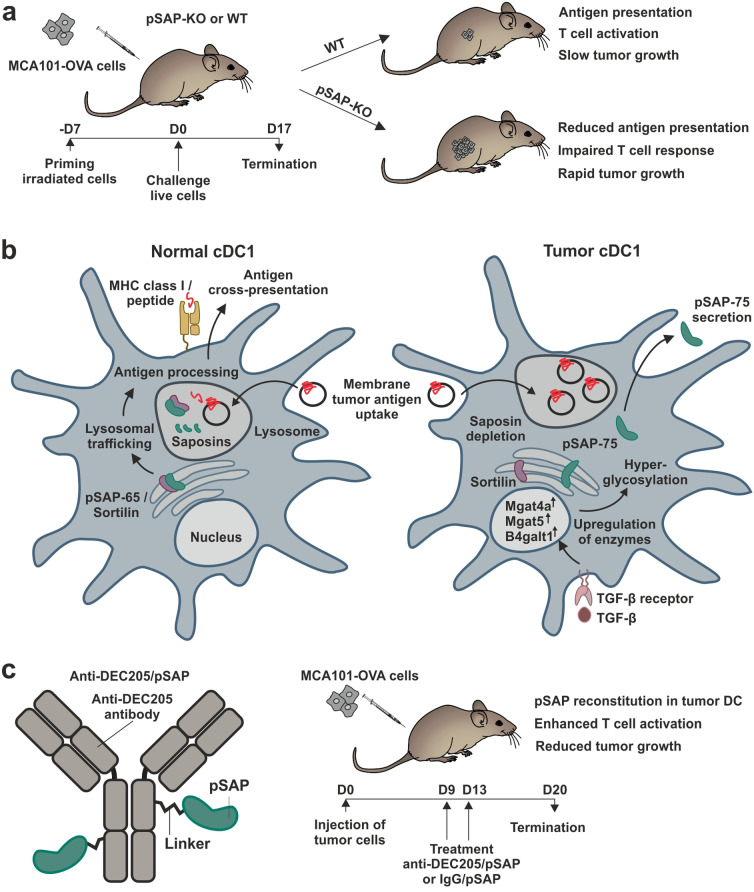
Impaired tumor immunity by prosaposin (pSAP) hyperglycosylation. **a** pSAP-KO or wildtype (wt) mice were primed and challenged with MCA101-OVA tumor cells expressing a membrane-associated form of ovalbumin (OVA). pSAP deficiency led to reduced antigen presentation, impaired T cell responses and rapid tumor growth. **b** Predicted mechanistic model: in normal cDC1, pSAP exists as the pSAP-65 form, which interacts with sortilin and is trafficked to the lysosome. Single saposins are generated and promote antigen processing, leading to cross-presentation on MHC class I. In tumor cDC1, transforming growth factor (TGF)-β promotes expression of enzymes involved in the generation of complex glycan structures including *N*-acetylglucosaminyltransferases Mgat4a and Mgat5 and galactosyltransferase B4galt1. pSAP is hyperglycosylated (pSAP-75), does not interact with sortilin and is secreted. Digestion of membranes is hampered leading to apoptotic body accumulation and impaired antigen presentation. **c** pSAP was chemically conjugated to a DEC205 antibody. In mice harboring MCA191-OVA tumors anti-DEC205/pSAP enhanced T cell activation and reduced tumor growth in comparison to treatment with an IgG/pSAP control conjugate. Parts of the Figure were generated using templates (DOIs 10.5281/zenodo.3925921 and 10.5281/zenodo.4912419) from SciDraw (https://scidraw.io)

P. Sharma and colleagues describe a novel mechanism by which tumors disturb cross-presentation of membrane antigens. Cross-presentation of membrane antigens involves lysosomes, in which extracellular antigens are processed and loaded onto MHC class I. In this context, a crucial function for saposins was uncovered. Saposins comprise five members (saposins A–D and GM2 activator protein), of which saposins A–D are generated by proteolytic cleavage of the precursor pSAP. Also engulfed tumor apoptotic bodies are trafficked into lysosomes, where after antigen processing tumor peptides are loaded onto MHC class I molecules to facilitate cross-priming.^1^ Comparing DC from pSAP-knockout (KO) and wildtype (WT) mice the authors found that pSAP expression was required for apoptotic body processing and cross-presentation of dead cell-associated antigens, but neither for antigen uptake nor for presentation of soluble antigens. Thus, pSAP-deficient DC pulsed with apoptotic murine MCA101-OVA fibrosarcoma cells ectopically expressing a membrane-associated form of ovalbumin (OVA) were inefficient in activating OVA-specific CD8^+^ T cells. In tumor challenge experiments increased tumor growth was observed in pSAP-KO mice (Fig. 1a). This was accompanied with reduced antigen presentation by DC isolated from tumor, impaired tumor infiltration by antigen-specific T cells and reduced T cell cytokine production. Yet, pSAP did not impact DC migration and infiltration. To reveal the role of pSAP in humans, CD11b/c^+^ myeloid cells (as a source of DC), CD8^+^ T cells and melanoma cells were sorted by flow cytometry from patient tumor samples. Myeloid cells were pulsed with irradiated melanoma cells and cocultured with autologous CD8^+^ T cells either in the presence or in the absence of recombinant pSAP. As a result, pSAP increased numbers of CD8^+^ T cells recognizing melanoma-associated antigens and enhanced IFN-γ production and degranulation.

The authors unraveled that tumor DC preferentially expressed a hyperglycosylated 75 kDa form of pSAP (pSAP-75) harboring a complex glycan containing *N*-acetylglucosamine, galactose, and sialic acid residues (Fig. 1b). Hyperglycosylated pSAP was specific to tumor DC. In splenic DC, pSAP-65 was predominant, of which glycan mainly contained mannose residues. Accordingly, in tumor DC several glycosyltransferases involved in maturation of complex glycans were upregulated. Interestingly, tumor DC were depleted of saposins and pSAP-75 was secreted. As an explanation, pSAP in both tumor DC from mice and melanoma-associated human DC did not interact with the chaperone sortilin, which is essential for lysosomal delivery of pSAP. In agreement, dominant expression of pSAP-75 with impaired interaction with sortilin was found in human DC isolated from the tumor-site from melanoma patients.

Notably, pSAP hyperglycosylation and secretion was triggered by transforming growth factor-β (TGF-β; Fig. 1b), which in vitro induced a glycosyltransferase signature resembling that of tumor DC. In mice with DC-specific deletion of TGF-β receptor II, enhanced tumor protection, increased antigen presentation in tumor DC and improved IFN-γ production by tumor infiltrating CD8^+^ T cells were observed. Tumor DC lacking TGF-β receptor II did not express pSAP-75 and contained single saposins in contrast to WT tumor DC and had alterations in the expression of individual glycosyltransferases and glycosidases.

Finally, pSAP was conjugated to an antibody targeting DEC-205, an endocytic type I C-type lectin receptor expressed by DC, for antigen-specific delivery (Fig. 1c). Treatment of pSAP-KO DC pulsed with apoptotic MCA101-OVA cells with this conjugate reconstituted antigen presentation and CD8^+^ T cell priming. In vivo, anti-DEC205/pSAP was taken up efficiently by tumor DC, reduced growth of MCA101-OVA tumor cells, enhanced presentation of OVA peptide and increased the frequency of IFN-γ-producing antigen specific T cells. Moreover, anti-DEC205/pSAP was evaluated in combination with PD-L1 immune checkpoint inhibition in the immunologically cold B16F10 melanoma model. While both single agents were not effective, combination treatment triggered CD8^+^ T cell activation and cytokine production, increased frequencies of specific T cells and reduced tumor growth.

These findings highlight an important role of saposins in the cross-presentation of corpuscular antigens by tumor DC to induce anti-tumoral T cell responses. The results add on to the recent observation that gelsolin impairs tumor immunity by inhibiting cross-presentation of cell-associated antigens.^5^ The authors unraveled that TGF-β induced pSAP hyperglycosylation was the underlying molecular mechanism leading to the depletion of saposins, thereby identifying a novel immune suppressive function for TGF-β. In this aspect the question arises, whether TGF-β also changes the glycan structure of other proteins and which consequences this may have. In the tumor microenvironment, TGF-β is secreted by various cell types, including cancer cells, cancer-associated fibroblasts or endothelial cells as well as different immune cells. The authors moreover suggested a novel therapeutic approach using an anti-DEC205/pSAP conjugate. Yet, although anti-DEC205/pSAP demonstrated efficacy, further studies will be required to assess feasibility in humans, taking differences in the expression pattern of DEC-205 between mice and humans into account. In addition, the situation may be more complex in cancer patients than in experimental mouse models. Immune escape involves various immune cell populations and pathways that contribute in establishing an immune hostile tumor micro-environment. Importantly, anti-DEC205/pSAP rendered resistant tumors responsive to PD-L1 antibody treatment, indicating that impaired antigen presentation by pSAP modulation impacts PD1/PD-L1 therapies and that pSAP reconstitution may allow overcoming resistance. It will be interesting to see, if this approach is effective also in models of other tumor types, especially in tumors with low mutational burden. Collectively, these findings are an important step forward in understanding immune evasion by tumors and may open new avenues for therapeutic intervention.
